# Sex Differences in the Associations of Obesity With Hypothyroidism and Thyroid Autoimmunity Among Chinese Adults

**DOI:** 10.3389/fphys.2018.01397

**Published:** 2018-10-04

**Authors:** Bin Wang, Ronghua Song, Weiwei He, Qiuming Yao, Qian Li, Xi Jia, Jin-an Zhang

**Affiliations:** ^1^Department of Endocrinology, Jinshan Hospital of Fudan University, Shanghai, China; ^2^Department of Endocrinology & Rheumatology, Shanghai University of Medicine & Health Sciences Affiliated Zhoupu Hospital, Shanghai, China; ^3^Department of Endocrinology, Affiliated Hospital of Yanan Medical University, Shaanxi, China

**Keywords:** obesity, hypothyroidism, thyroid autoimmunity, risk factor, thyroid dysfunction

## Abstract

There is an intensive link between obesity and thyroid dysfunction, but this relationship in Asians is still unclear. This study was conducted to define the impact of obesity on risk of hypothyroidism and thyroid autoimmunity among Chinese adults. A population-based, cross-sectional study was carried out, which enrolled a total of 2,808 Chinese adults. To assess the associations of obesity with hypothyroidism and thyroid autoimmunity, odds ratio (ORs) with 95% confidence intervals (95%CIs) were calculated through logistic regression model, and the correlations of body mass index (BMI) with TPOAb and TGAb were also analyzed. Obese females had higher risk of hypothyroidism (22.7 vs. 15.0%; OR = 1.66, 95%CI 1.10–2.53; *P* = 0.02) and higher risk of subclinical hypothyroidism (22.1 vs. 13.4%; OR = 1.83, 95%CI 1.20–2.80; *P* = 0.005) than non-obese females. Multivariate logistic regression analysis found significant associations of obesity with hypothyroidism (Adjusted OR = 1.54, 95%CI 1.00–2.38; *P* = 0.05) and subclinical hypothyroidism (Adjusted OR = 1.69, 95%CI 1.09–2.63; *P* = 0.02) in females after adjustment for confounding factors. No association between obesity and hypothyroidism was observed in male participants. Spearman's correlation analysis suggested BMI was significantly and positively correlated with TPOAb (Spearman's r = 0.062, *P* = 0.022) in men but not in women. Linear regression analysis suggested an obviously positive correlation of BMI with TPOAb in men (β = 0.018, *P* = 0.015) and an obviously negative correlation of BMI with TGAb in women (β = −0.025, *P* = 0.012), respectively. The study suggests sex differences in the associations of obesity with hypothyroidism and thyroid autoimmunity among Chinese adults. Further studies are needed to better understand the exact mechanism of sex difference in the obesity-thyroid relationship.

## Introduction

The epidemic of obesity has become a major health problem worldwide in the past decade (Bray et al., 2017). With the rapidly increasing prevalence, obesity and its related diseases have caused increasing harm to public health (Flegal et al., 2016; Afshin et al., 2017). There are over two billion overweight or obese adults worldwide (Malik et al., 2013; Risk Factor Collaboration (NCD-RisC), 2017). Epidemiological studies have proven that obesity is an important risk factor of many diseases, such as stroke, coronary heart disease, cancer and diabetes (Mitchell et al., 2015; Afshin et al., 2017; Kivimaki et al., 2017). Obesity is also independently related to increased risk of all-cause mortality (Flegal et al., 2013).

Thyroid disorders are highly prevalent diseases of the endocrine system, and mainly include thyroid dysfunction and autoimmune thyroid diseases (AITDs) (Visser et al., 2013; Tomer, 2014). Thyroid dysfunction mainly includes hyperthyroidism and hypothyroidism (Taylor et al., 2018). AITDs are common autoimmune diseases and are the principal cause of thyroid dysfunction, which mainly include Hashimoto's thyroiditis (HT) and Graves' disease (GD) (Antonelli et al., 2015). Both HT and GD are characterized by immune imbalance of immune cells and aberrant expressions of key cytokines (Antonelli et al., 2015). Thyroid autoimmunity (TAI), defined as positivity for thyroid antibodies, has gained increasing attention as its high prevalence in the general population and its adverse effect on human health (Wiersinga, 2014). Both thyroid dysfunction and TAI have obviously adverse impact on human health and can lead to increased risk of cardiovascular diseases and mortality (Baumgartner et al., 2017; Journy et al., 2017; Martin et al., 2017).

The influence of obesity on thyroid dysfunction, such as hypothyroidism has been studied in several epidemiological studies in Western countries (Rimm et al., 1975; Asvold et al., 2009; Gopinath et al., 2010; Marzullo et al., 2010; Ittermann et al., 2013; Garcia-Garcia et al., 2016). Some studies found that obesity could increase risk of hypothyroidism (Rimm et al., 1975; Asvold et al., 2009; Gopinath et al., 2010; Marzullo et al., 2010). On the contrary, other studies reported no increased risk of hypothyroidism or subclinical hypothyroidism (SCH) among obese patients (Ittermann et al., 2013; Garcia-Garcia et al., 2016). Therefore, there is still lack of a definite conclusion on the association of obesity with hypothyroidism, and studies from Asians are still lacking. Recent studies suggested that obesity could increase the risk of autoimmune diseases, such as inflammatory bowel disease, psoriatic arthritis and rheumatoid arthritis, suggesting a potential link between obesity and autoimmunity (Love et al., 2012; Gremese et al., 2014; Khalili et al., 2015; Ljung and Rantapaa-Dahlqvist, 2016). While the impact of obesity on thyroid dysfunction has been evaluated by several observational studies, the relationship between obesity and TAI remains undetermined. Bedsides, the prevalence of hypothyroidism and TAI is various across different ethnic populations (Wiersinga, 2014), and the impact of obesity on hypothyroidism and TAI among the Chinese population may be different from other populations. To our knowledge, no study has evaluated the impact of obesity on risk of hypothyroidism and TAI among Chinese adults. Therefore, we conducted a population-based, cross-sectional study to define the influence of obesity on the risk of hypothyroidism and TAI among Chinese adults.

## Methods

### Participants

This cross-sectional study was conducted in 2016 in Shanghai, China. A total of 2,824 individuals were randomly selected from the general population and were preliminarily enrolled, and 16 with a history of thyroidectomy for thyroid cancer were excluded. Therefore, 2,808 eligible individuals [1,379 males and 1,429 females) were enrolled in this study. All of those participants were from the Chinese Han population. All subjects gave written informed consent in accordance with the Declaration of Helsinki. The protocol was approved by the Ethics Committee of Jinshan Hospital of Fudan University.

### Clinical examination

Questionnaires on information of age, sex, occupation, smoking, type of salt intake, prior disease history and use of medications were completed by all participants. Height, body weight and waist circumference of all participants were measured by experienced physicians. Body mass index (BMI) was determined as weight in kilograms divided by the square of height in meters (kg/m^2^). Overweight was defined as 24 ≤ BMI < 28 kg/m^2^, and obesity was defined as BMI ≥ 28 kg/m^2^ (Zhou, 2002). Lean body weight was as BMI < 18.5 kg/m^2^ (Zhou, 2002).

### Laboratory testing

Fasting blood samples were drawn between 7:00 and 10:00 after fasting overnight. Thyroid hormones and thyroid antibodies including thyroid stimulating hormone (TSH), free thyroxine (fT4), free triiodothyronine (fT3), thyroid peroxidase antibody (TPOAb) and thyroglobulin antibody (TGAb) were quantified using immunochemiluminometric assay (ICMA) method. The determinations of thyroid hormones and thyroid antibodies were performed in the same laboratory in the First Hospital of China Medical University. The intra-assay coefficient of variation and the inter-assay coefficient of variation for serum TSH were 1.57–4.12% and 1.26–5.76%, respectively (Yu et al., 2015; Teng et al., 2018). To screen the prevalence of thyroid disorders in the population, TSH and thyroid antibodies were measured in all participants, but fT4 and fT3 were measured only among those with abnormal levels of TSH. Urinary iodine concentration was quantified using the ammonium persulfate method. The reference values were 0.27–4.2 mIU/L for TSH, 12–22 pmol/L for fT4, and 3.1–6.8 pmol/L for fT3. The reference values were 0–34 IU/L for TPOAb and 0–50 IU/L for TGAb. For TPOAb and TGAb, positivity was set at >34 and >50 IU/L, respectively.

### Hypothyroidism and thyroid autoimmunity

TAI was described as either positivity for TPOAb or positivity for TGAb. Hypothyroidism in this cross-sectional study included overt hypothyroidism and SCH. Overt hypothyroidism was defined by decreased fT4 with increased TSH level, or thyroid hormone replacement therapy for hypothyroidism. SCH was defined as increased TSH level with normal fT4 level.

### Statistical analysis

Continuous variables with normal distribution were presented as mean with standard deviation (SD), and the *t*-test was used to compare between-group difference. Continuous variables with skewed distribution were presented as median with interquartile range (IQR), and Mann-Whitney U test was used to compare between-group difference. Categorical variables were presented as frequencies with percentages, and Chi-square test was used to compare between-group difference. The prevalence of hypothyroidism, TAI, positive TPOAb and positive TGAb were firstly compared between obese individuals and non-obese individuals, and then was compared between obese individuals and individuals with normal weight. Stratified analyses were conducted by types of hypothyroidism, sex, age of participants (Ages < 65 years vs. ages ≥65 years) and diabetes status (Diabetic participants vs. Non-diabetic participants). For overt hypothyroidism, the low number of participants precluded adequate estimates, and the results were therefore omitted. To obtain the odds ratio (ORs) with 95% confidence intervals (95%CIs), logistic regression method was used. Age, sex, smoking, diabetes, BMI, hypertension, type of salt intake and urinary iodine concentration were used as covariates in the multivariate logistic regression analysis. To further assess the impact of obesity on TAI, the correlations of BMI with thyroid antibodies were analyzed using Spearman's correlation analysis since the values of thyroid antibodies were skewedly distributed. Relationships of BMI with thyroid antibodies were also assessed through linear regression analysis, and confounding factors included age, sex, smoking, diabetes, BMI, hypertension, type of salt intake and urinary iodine concentration. Values of thyroid antibodies were skewedly distributed, and were log2-transformed in the linear regression analysis. STATA (Version 12.0, StataCorp, USA) was used to conduct statistics, and *P*-value < 0.05 was considered statistically significant.

## Results

### Characteristics of participants

The demographic and clinical features of participants were summarized in Table 1 (Table 1). Those 2,808 individuals included 1,379 men and 1,429 women. The age of participants ranged from 18 to 89 years, with a mean of 43.6 years. Among them, 397 (14.1%) were categorized to have obesity, 943 (33.6%) with overweight, 1,366 (48.6%) with normal weight, and 102 (3.6%) with lean body weight. The prevalence of obesity was higher in men (18.3%) than women (10.1%) in this population (*P* < 0.001; Table 1). Men had a higher prevalence of type 2 diabetes (*P* = 0.003) and hypertension (*P* < 0.001) than women (Table 1). Meanwhile, women had higher levels of TSH (*P* < 0.001), TGAb (*P* < 0.001) and TPOAb (*P* < 0.001) than men (Table 1). There were 354 (12.6%) hypothyroidism individuals including 326 SCH (11.6%) and 28 (1.0%) overt hypothyroidism. Among all participants, 359 (12.8%) were found to have TAI, containing 266 (9.5%) TPOAb positivity and 308 (10.9%) TGAb positivity. Table 2 showed the comparison of clinical characteristics between obese individuals and non-obese controls (Table 2). Compared with those non-obese controls, obese individuals had higher frequencies of smoking, type 2 diabetes and hypertension (*P* < 0.001; Table 2). However, obese individuals did not have increased levels of TSH, TPOAb, and TGAb when compared with non-obese controls (*P* > 0.05, Table 2).

**Table 1:** Clinical characteristics of study subjects in the cross-sectional study.

**Characteristics**	**All**	**Men**	**Women**	***P***
Number	2,808	1,379	1,429	
Age (range, years)	18–89	18–87	18–89	–
Mean age (years)	43.6 (15.8)	42.8 (16.3)	44.3 (15.3)	0.01
BMI (kg/m^2^)	24.2 (10.0)	24.9 (3.9)	23.5 (3.6)	< 0.001
Obesity [*n*(%)]	397 (14.1%)	252 (18.3%)	145 (10.1%)	< 0.001
Smoking [*n*(%)]	752 (26.8%)	731 (53.0%)	21 (1.5%)	< 0.001
Type 2 diabetes [*n*(%)]	369 (13.1%)	208 (15.1%)	161 (11.3%)	0.003
Hypertension [*n*(%)]	869 (30.9%)	514 (37.3%)	355 (24.8%)	< 0.001
Iodized salt intake [*n*(%)]	2,179 (77.6%)	1,116 (80.9%)	1,063 (74.4%)	< 0.001
Urinary iodine concentration	164.5 (111.1–235.6)	171.3 (121.6–248.7)	155.5 (100.9–225.8)	< 0.001
TSH (mIU/l)	2.4 (1.7–3.3)	2.2 (1.6–2.9)	2.6 (1.8–3.5)	< 0.001
TPOAb (U/ml)	8.8 (6.4–12.6)	8.4 (6.4–11.9)	9.1 (6.5–13.8)	< 0.001
TGAb (U/ml)	11.7 (10.0–15.2)	11.6 (10.0–14.2)	11.9 (10.0–16.9)	< 0.001

*Normally distributed variables were expressed as mean ± SD, non-normally distributed variables as median with IQR, and categorical variables as number with percentages (%). BMI, body mass index; TSH, thyroid stimulating hormone; TPOAb, thyroid peroxidase antibody; TGAb, thyroglobulin antibody*.

**Table 2:** Comparison of clinical characteristics between obese individuals and non-obese controls.

**Characteristics**	**Obese individuals**	**Non-obese controls**	***P***
Number	*N* = 397	*N* = 2,411	
Age (range, years)	18–89	18–87	–
Mean age (years)	44.3 (16.3)	43.4 (15.8)	0.28
BMI (kg/m^2^)	30.7 (2.7)	23.1 (2.7)	< 0.001
Smoking [*n* (%)]	144 (36.3%)	608 (25.2%)	< 0.001
Type 2 diabetes [*n* (%)]	87 (21.9%)	282 (11.7%)	< 0.001
Hypertension [*n* (%)]	225 (56.7%)	644 (26.7%)	< 0.001
Iodized salt intake [*n* (%)]	314 (79.1%)	1865(77.4%)	0.441
Urinary iodine concentration	160.0 (114.6–227.9)	165.1 (110.5–237.4)	0.569
TSH (mIU/l)	2.39 (1.69–3.27)	2.34 (1.71–3.29)	0.622
TPOAb (U/ml)	8.41 (6.47–12.40)	8.83 (6.41–12.62)	0.349
TGAb (U/ml)	12.05 (10.00–15.05)	11.69 (10.00–15.18)	0.609

*Normally distributed variables were expressed as mean ± SD, non-normally distributed variables as median with IQR, and categorical variables as number with percentages (%). BMI, body mass index; TSH, thyroid stimulating hormone; TPOAb, thyroid peroxidase antibody; TGAb, thyroglobulin antibody*.

### Obesity and hypothyroidism

**Figure 1:**
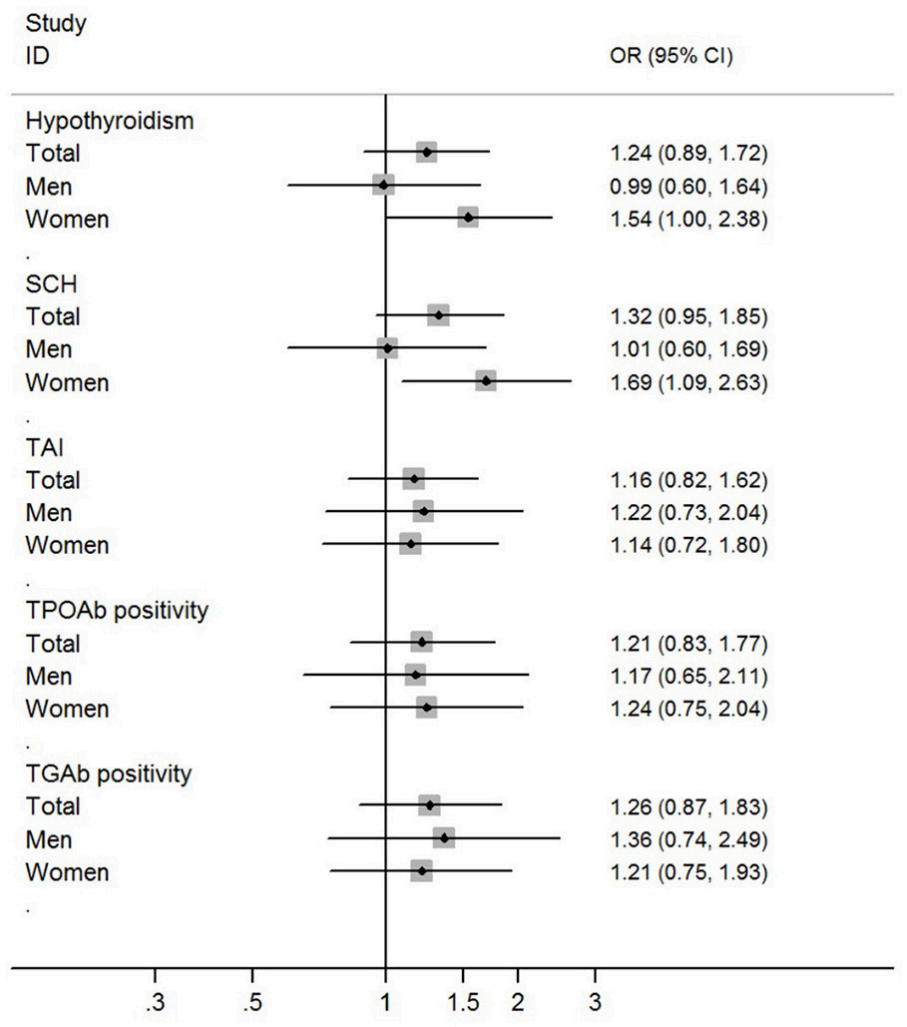
Risk of hypothyroidism and thyroid autoimmunity among obese individuals when compared with non-obese individuals.

The prevalence of hypothyroidism was not higher in obese patients than non-obese controls (13.9 vs. 12.4%; *P* = 0.42). The prevalence of SCH was also not higher in obese patients than non-obese controls (13.3 vs. 11.3%; *P* = 0.24). Multivariate logistic regression analysis didn't find significant associations of obesity with hypothyroidism and SCH (Table 3; Figure 1).

**Table 3:** Risk of hypothyroidism and thyroid autoimmunity among obese individuals compared with non-obese individuals.

**Thyroid dysfunction**	**Obese individuals**	**Non-obese controls**	**Crude OR**		Adjusted OR^*^	
			**OR (95%CI)**	***P***	**OR (95%CI)**	***P***
Total	*N* = 397	*N* = 2,411				
Hypothyroidism^#^	55 (13.9%)	299 (12.4%)	1.14 (0.83–1.55)	0.42	1.24 (0.89–1.72)	0.20
SCH	53 (13.3%)	273 (11.3%)	1.21 (0.88–1.65)	0.24	1.32 (0.95–1.85)	0.10
TAI	49 (12.3%)	310 (12.8%)	0.95 (0.69–1.32)	0.78	1.16 (0.82–1.62)	0.40
TPOAb positivity	38 (9.6%)	228 (9.5%)	1.01 (0.71–1.45)	0.94	1.21 (0.83–1.77)	0.32
TGAb positivity	42 (10.6%)	266 (11.0%)	0.95 (0.68–1.35)	0.79	1.26 (0.87–1.83)	0.21
Men	*N* = 252	*N* = 1,127				
Hypothyroidism^#^	22 (8.7%)	106 (9.4%)	0.92 (0.57–1.49)	0.74	0.99 (0.60–1.64)	0.97
SCH	21 (8.3%)	101 (8.9%)	0.92 (0.56–1.51)	0.75	1.01 (0.60–1.69)	0.97
TAI	22 (8.7%)	83 (7.4%)	1.20 (0.74–1.97)	0.46	1.22 (0.73–2.04)	0.44
TPOAb positivity	16 (6.3%)	55 (4.9%)	1.16 (0.66–2.05)	0.60	1.17 (0.65–2.11)	0.61
TGAb positivity	17 (6.7%)	52 (4.6%)	1.38 (0.79–2.42)	0.26	1.36 (0.74–2.49)	0.32
Women	*N* = 145	*N* = 1,284				
Hypothyroidism^#^	33 (22.7%)	193 (15.0%)	**1.66 (1.10–2.53)**	**0.02**	**1.54 (1.00–2.38)**	**0.05**
SCH	32 (22.1%)	172 (13.4%)	**1.83 (1.20–2.80)**	**0.005**	**1.69 (1.09–2.63)**	**0.02**
TAI	27 (18.6%)	227 (17.7%)	1.06 (0.68–1.66)	0.78	1.14 (0.72–1.80)	0.57
TPOAb positivity	22 (15.2%)	150 (11.7%)	1.20 (0.74–1.95)	0.45	1.24 (0.75–2.04)	0.40
TGAb positivity	25 (17.2%)	195 (15.2%)	1.06 (0.67–1.68)	0.79	1.21 (0.75–1.93)	0.43

* *Confounding factors in the multiple logistic regression analysis included age, sex, smoking, diabetes, hypertension, salt type and urinary iodine concentration*.

# *Hypothyroidism included SCH and overt hypothyroidism. OR, odds ratio; 95%CI, 95% confidence interval. Bold values were statistically significant outcomes*.

In the stratified analysis by sex, a higher prevalence of hypothyroidism was found in female obese patients than female non-obese controls (22.7 vs. 15.0%; OR = 1.66, 95%CI 1.10–2.53; *P* = 0.02). A higher prevalence of SCH was also found in female obese patients than female non-obese controls (22.1 vs. 13.4%; OR = 1.83, 95%CI 1.20–2.80; *P* = 0.005). Multivariate logistic regression analysis found independent and significant associations of obesity with hypothyroidism (Adjusted OR = 1.54, 95%CI 1.00–2.38; *P* = 0.05) and SCH (Adjusted OR = 1.69, 95%CI 1.09–2.63; *P* = 0.02) in female participants (Table 3; Figure 1). When using individuals with normal weight as controls, there were significant associations of obesity with hypothyroidism (OR = 1.72, 95%CI 1.11–2.65; *P* = 0.015) and SCH (OR = 1.84, 95%CI 1.19–2.86; *P* = 0.007) among female participants (Table 4).

**Table 4:** Risk of hypothyroidism and thyroid autoimmunity among obese individuals compared with individuals with normal weight.

**Thyroid dysfunction**	**Obese individuals**	**Normal weight**	**Crude OR**		Adjusted OR^*^	
			**OR (95%CI)**	***P***	**OR (95%CI)**	***P***
Total	*N* = 397	*N* = 1,366				
Hypothyroidism^#^	55 (13.9%)	170 (12.4%)	1.13 (0.82–1.57)	0.46	1.25 (0.86–1.80)	0.24
SCH	53 (13.3%)	157 (11.5%)	1.19 (0.85–1.66)	0.32	1.31 (0.90–1.91)	0.16
TAI	49 (12.3%)	181 (13.3%)	0.92 (0.66–1.29)	0.64	1.11 (0.76–1.61)	0.60
TPOAb positivity	38 (9.6%)	114 (8.3%)	1.03 (0.71–1.51)	0.87	1.24 (0.81–1.91)	0.32
TGAb positivity	42 (10.6%)	148 (10.8%)	0.90 (0.63–1.30)	0.58	1.29 (0.86–1.94)	0.22
Men	*N* = 252	*N* = 540				
Hypothyroidism^#^	22 (8.7%)	49 (%)	0.96 (0.57–1.62)	0.87	1.01 (0.56–1.80)	0.98
SCH	21 (8.3%)	47 (9.1%)	0.95 (0.56–1.63)	0.86	1.01 (0.56–1.83)	0.97
TAI	22 (8.7%)	34 (6.3%)	1.42 (0.81–2.49)	0.21	1.27 (0.68–2.35)	0.45
TPOAb positivity	16 (6.3%)	19 (3.5%)	1.52 (0.79–2.94)	0.21	1.30 (0.63–2.68)	0.48
TGAb positivity	17 (6.7%)	22 (4.1%)	1.56 (0.82–2.95)	0.18	1.54 (0.75–3.19)	0.24
Women	*N* = 145	*N* = 826				
Hypothyroidism^#^	33 (22.7%)	121 (14.6%)	**1.72 (1.11–2.65)**	**0.015**	1.50 (0.93–2.42)	0.09
SCH	32 (22.1%)	110 (13.3%)	**1.84 (1.19–2.86)**	**0.007**	**1.63 (1.00–2.65)**	**0.048**
TAI	27 (18.6%)	147 (17.8%)	1.06 (0.67–1.66)	0.81	1.01 (0.62–1.65)	0.96
TPOAb positivity	22 (15.2%)	95 (11.5%)	1.24 (0.75–2.04)	0.39	1.16 (0.68–2.00)	0.58
TGAb positivity	25 (17.2%)	126 (15.3%)	1.08 (0.67–1.72)	0.76	1.18 (0.71–1.95)	0.52

* *Confounding factors in the multiple logistic regression analysis included age, sex, smoking, diabetes, hypertension, salt type and urinary iodine concentration*.

# *Hypothyroidism included SCH and overt hypothyroidism. OR, odds ratio; 95%CI, 95% confidence interval. Bold values were statistically significant outcomes*.

In the stratified analysis of male participants, there was no difference in the prevalence of hypothyroidism and SCH between obese patients and non-obese controls (Table 3; Figure 1). Multivariate logistic regression analysis also didn't find any significant association of obesity with hypothyroidism and SCH among male participants (Table 3; Figure 1). When using individuals with normal weight as controls, there was no obvious association of obesity with hypothyroidism and SCH among male participants (Table 4).

In participants aged 65 years or more, there was a higher prevalence of hypothyroidism in female obese patients than female non-obese controls (37.9 vs. 19.8%; OR = 2.47, 95%CI 1.04–5.86; *P* = 0.04), but it was not statistically significant after adjustment for confounding factors (Table 5). In participants aged < 65 years, there was no obvious difference in the prevalence of hypothyroidism or SCH between obese patients and non-obese controls (Table 5).

**Table 5:** Risk of hypothyroidism and thyroid autoimmunity among obese individuals compared with non-obese individuals in the subgroup analyses by age.

**Thyroid dysfunction**	**Obese individuals**	**Non-obese controls**	**Crude OR**		Adjusted OR^*^	
			**OR (95%CI)**	***P***	**OR (95%CI)**	***P***
**AGES**<**65 YEARS**						
Total	*N* = 342	*N* = 2,150				
Hypothyroidism^#^	40 (11.7%)	250 (11.6%)	1.01 (0.70–1.43)	0.97	1.20 (0.83–1.75)	0.33
SCH	38 (11.1%)	227 (10.6%)	1.06 (0.73–1.52)	0.76	1.27 (0.86–1.86)	0.23
TAI	37 (10.8%)	273 (12.7%)	0.83 (0.58–1.20)	0.33	1.04 (0.71–1.53)	0.82
TPOAb positivity	32 (9.4%)	197 (9.2%)	0.95 (0.64–1.42)	0.82	1.17 (0.77–1.78)	0.46
TGAb positivity	32 (9.4%)	238 (11.1%)	0.83 (0.56–1.22)	0.34	1.16 (0.77–1.76)	0.46
Men	*N* = 226	*N* = 997				
Hypothyroidism^#^	18 (7.9%)	83 (8.3%)	0.95 (0.56–1.62)	0.86	1.00 (0.57–1.76)	0.99
SCH	17 (7.5%)	81 (8.1%)	0.92 (0.53–1.58)	0.76	0.98 (0.55–1.75)	0.96
TAI	17 (7.5%)	72 (7.2%)	1.04 (0.60–1.81)	0.87	1.03 (0.58–1.83)	0.92
TPOAb positivity	14 (6.2%)	54 (5.4%)	1.15 (0.63–2.11)	0.64	1.17 (0.62–2.21)	0.63
TGAb positivity	12 (5.3%)	48 (4.8%)	1.11 (0.58–2.12)	0.76	1.07 (0.54–2.13)	0.84
Women	*N* = 116	*N* = 1,153				
Hypothyroidism^#^	22 (18.9%)	167 (14.5%)	1.38 (0.84–2.26)	0.19	1.44 (0.87–2.38)	0.16
SCH	21 (18.1%)	146 (12.7%)	1.52 (0.92–2.52)	0.10	1.62 (0.98–2.72)	0.06
TAI	20 (17.2%)	201 (17.4%)	0.99 (0.59–1.63)	0.96	1.05 (0.62–1.76)	0.86
TPOAb positivity	18 (15.5%)	143 (12.4%)	1.13 (0.65–1.97)	0.67	1.20 (0.68–2.12)	0.53
TGAb positivity	20 (17.2%)	190 (16.5%)	1.05 (0.64–1.75)	0.83	1.17 (0.69–1.96)	0.56
**AGES** ≥**65 YEARS**						
Total	*N* = 55	*N* = 261				
Hypothyroidism^#^	15 (27.3%)	49 (18.8%)	1.62 (0.83–3.17)	0.16	1.58 (0.79–3.17)	0.20
SCH	15 (27.3%)	46 (17.6%)	1.75 (0.89–3.43)	0.10	1.71 (0.85–3.45)	0.13
TAI	12 (21.8%)	37 (14.2%)	1.69 (0.81–3.49)	0.16	1.85 (0.86–3.98)	0.11
TPOAb positivity	8 (14.5%)	31 (11.9%)	1.26 (0.55–2.92)	0.58	1.25 (0.52–3.00)	0.62
TGAb positivity	10 (18.2%)	28 (10.7%)	1.85 (0.84–4.07)	0.13	2.23 (0.95–5.22)	0.06
Men	*N* = 26	*N* = 130				
Hypothyroidism^#^	4 (15.4%)	23 (17.7%)	0.84 (0.27–2.69)	0.78	1.03 (0.30–3.48)	0.96
SCH	4 (15.4%)	20 (15.4%)	1.00 (0.31–3.21)	0.99	1.21 (0.36–4.12)	0.76
TAI	5 (19.2%)	11 (8.5%)	2.57 (0.81–8.17)	0.11	2.55 (0.75–8.74)	0.13
TPOAb positivity	2 (7.7%)	8 (6.1%)	1.27 (0.25–6.36)	0.77	1.12 (0.21–6.07)	0.89
TGAb positivity	5 (19.2%)	8 (6.1%)	**3.63 (1.08–12.17)**	**0.04**	**4.28 (1.09–16.78)**	**0.03**
Women	*N* = 29	*N* = 131				
Hypothyroidism^#^	11 (37.9%)	26 (19.8%)	**2.47 (1.04–5.86)**	**0.04**	2.16 (0.87–5.37)	0.09
SCH	11 (37.9%)	26 (19.8%)	**2.47 (1.04–5.86)**	**0.04**	2.16 (0.87–5.37)	0.09
TAI	7 (24.1%)	26 (19.8%)	1.28 (0.49–3.33)	0.61	1.53 (0.56–4.15)	0.41
TPOAb positivity	6 (20.7%)	23 (17.5%)	1.22 (0.45–3.35)	0.69	1.28 (0.45–3.63)	0.64
TGAb positivity	5 (17.2%)	20 (15.3%)	1.16 (0.39–3.39)	0.79	1.41 (0.45–4.41)	0.55

* *Confounding factors in the multiple logistic regression analysis included age, sex, smoking, diabetes, hypertension, salt type and urinary iodine concentration*.

# *Hypothyroidism included SCH and overt hypothyroidism. OR, odds ratio; 95%CI, 95% confidence interval. Bold values were statistically significant outcomes*.

In non-diabetic participants, there was a higher prevalence of SCH in female obese patients than female non-obese controls (21.1 vs. 13.5%; OR = 1.71, 95%CI 1.04–2.78; *P* = 0.03), but it was not statistically significant after adjustment for confounding factors (Supplementary Table 1). In diabetic participants, there was no obvious difference in the prevalence of hypothyroidism or SCH between obese patients and non-obese controls (Supplementary Table 1).

### Obesity and TAI

There was no discernible difference in the prevalence of TAI between obese patients and non-obese controls (12.1 vs. 12.8%; OR = 0.93, 95%CI 0.67–1.29, *P* = 0.67; Table 3). Obesity was not linked to either TPOAb positivity or TGAb positivity among all participants (Table 3). Stratified analysis by sex also revealed that obesity was not related to TAI, TPOAb positivity and TGAb positivity in both men and women (Table 3). When using individuals with normal weight as controls, there was no significant association of obesity with TAI, TPOAb positivity and TGAb positivity (Table 4).

Among participants older than 65 years, there was a higher prevalence of TGAb positivity in male obese patients than male non-obese controls (19.2 vs. 6.1%; OR = 4.28, 95%CI 1.09–16.78; *P* = 0.03). In participants aged < 65, there was no obvious difference in the prevalence of TAI between obese patients and non-obese controls (Table 5). Stratified analysis by diabetes did not reveal any significant finding (Supplementary Table 1).

To further assess the impact of obesity on TAI, the correlations of BMI with concentrations of TPOAb and TGAb were analyzed using both Spearman's correlation analysis and linear regression analysis. Spearman's correlation analysis suggested that BMI was significantly and positively correlated with TPOAb (Spearman's r = 0.062, *P* = 0.022) in men but not in women (Spearman's r = 0.034, *P* = 0.201; Table 6). TGAb was marginally correlated with BMI in women (Spearman's r = −0.051, *P* = 0.053) but not in men (Spearman's r = 0.031, *P* = 0.258; Table 6). Linear regression analysis suggested an obviously positive correlation of BMI with TPOAb in men (regression coefficient β = 0.018, *P* = 0.015) but not in women (regression coefficient β = 0.005, *P* = 0.689), and an obviously negative correlation of BMI with TGAb in women (regression coefficient β = −0.025, *P* = 0.012) but not in men (regression coefficient β = 0.009, *P* = 0.245; Table 6). Multiple linear regression analysis further demonstrated an independently positive correlation of BMI with TPOAb in men (regression coefficient β = 0.016, *P* = 0.036; Table 6). Stratified analysis by age and diabetes also revealed some distinct correlations between BMI and thyroid autoimmunity among females and males (Supplementary Table 2). For instance, linear regression analysis suggested an obviously positive correlation of BMI with TPOAb in diabetic men (regression coefficient β = 0.045, *P* = 0.012) but not in diabetic women (regression coefficient β = 0.035, *P* = 0.289; Supplementary Table 2). However, there was an obviously negative correlation of BMI with TGAb in non-diabetic women (regression coefficient β = −0.034, *P* = 0.014) but not in non-diabetic men (regression coefficient β = 0.008, *P* = 0.338; Supplementary Table 2).

**Table 6:** Correlations between BMI and thyroid autoimmunity.

**Outcomes**	**Spearman's correlation analysis**		**Univariate linear regression analysis**		**Multiple linear regression analysis**	
	**r (SE)**	***P*-value**	**β (SE)**	***P*-value**	**β (SE)**	***P*-value**
**TOTAL**
TPOAb	0.033 (0.019)	0.083	0.002 (0.007)	0.782	0.005 (0.007)	0.445
TGAb	−0.028 (0.019)	0.131	–**0.018 (0.007)**	**0.010**	−0.005 (0.007)	0.490
**MEN**
TPOAb	**0.062 (0.028)**	**0.022**	**0.018 (0.007)**	**0.015**	**0.016 (0.008)**	**0.036**
TGAb	0.031 (0.028)	0.258	0.009 (0.007)	0.245	0.008 (0.008)	0.297
**WOMEN**
TPOAb	0.034 (0.027)	0.201	0.005 (0.012)	0.689	−0.007 (0.013)	0.600
TGAb	–**0.051 (0.027)**	**0.053**	–**0.025 (0.012)**	**0.041**	−0.022 (0.013)	0.104

*Values of thyroid antibodies were nonnormally distributed, and were log2-transformed in the linear regression analysis. SE, standard error. Bold values were statistically significant outcomes*.

## Discussion

Currently, the impact of obesity on hypothyroidism and TAI has not been characterized clearly, and few data from Asians are available. To our knowledge, this study is the first population-based, cross-sectional study investigating the impact of obesity on risk of hypothyroidism and TAI among Chinese adults. We found evidence of a significant association of obesity with hypothyroidism in women but not in men. Moreover, BMI was significantly correlated to TPOAb in men but not in women, while BMI was significantly correlated to TGAb in women but not in men. Therefore, the findings suggested sex differences in the associations of obesity with hypothyroidism and TAI among Chinese adults.

The sex differences in the associations of obesity with hypothyroidism and TAI may be related to sex differences in the body fat distribution between men and women, which may result in sex-specific changes in the peripheral metabolism of thyroid hormones (Laurberg et al., 2012; Santini et al., 2014; Fontenelle et al., 2016). Another possible explanation is the sex differences in the effects of sex hormones on thyroid dysfunction and TAI between men and women, since estradiol and testosterone exert different roles in regulating thyroid functions and immune response (Marqusee et al., 2000; Bahrami et al., 2009; Roberts et al., 2009; Laurberg et al., 2012; Khan and Ansar Ahmed, 2015; Chen et al., 2017; Habib et al., 2018). Obesity may cause different and aberrant changes in sex hormones between men and women, which may lead to the sex-specific risk of thyroid dysfunction and TAI. Finally, the sex-specific associations of obesity with hypothyroidism and TAI may arise from the differences in the adipokines between men and women (Wauters and Van Gaal, 1999; Aguado et al., 2008; Saltevo et al., 2009). Many adipokines, such as leptin and adiponectin, have important roles in regulating immunity and have been considered as pivotal links between obesity and obesity-related diseases, and the sex-specific roles of adipokines may thus be involved in the sex-specific associations of obesity with hypothyroidism and TAI (Wilk et al., 2011; Fantuzzi, 2013; Versini et al., 2014; Gerriets et al., 2016; Obeid et al., 2017). However, the underlying mechanisms remain largely unknown, and further researches are warranted to uncover them.

A comprehensive understanding of the impact of obesity on thyroid disorders is important to motivate people to take appropriate interventions to reduce thyroid disorders. Previous studies have established that obesity can significantly increase risk of thyroid cancer (Marcello et al., 2014; Pappa and Alevizaki, 2014; Xu et al., 2014), but the impact of obesity on thyroid dysfunction has not been clearly defined. Published epidemiological studies showed conflicting findings in the association of obesity with hypothyroidism (de Moura Souza and Sichieri, 2011; Laurberg et al., 2012; Poddar et al., 2017). The findings from our study suggested sex differences in the associations of obesity with hypothyroidism, and obesity was related to increased hypothyroidism risk in women but not in men. However, the causative relationship between obesity and hypothyroidism is still not well-defined, because most published epidemiological studies used cross-sectional or case-control design but not prospective cohort design, which had limited power to evaluate the exact role of obesity in the development of hypothyroidism. Therefore, more prospective cohort studies with well-design and large number of participants are needed to further define whether a causative link between obesity and hypothyroidism exists.

Studies in recent years have proven a high prevalence of thyroid dysfunction among obese patients, especially among those with extreme obesity, which suggests appropriate monitoring of thyroid function may be recommended among obese patients (Fontenelle et al., 2016; Niranjan and Wright, 2016). Some studies also suggested that obese patients with hypothyroidism had much poorer outcomes than those without thyroid dysfunction (Brienza et al., 2013; Wang et al., 2016). The elevated prevalence of hypothyroidism observed in obese patients may be explained by the increased thyroxine requirement caused by excess weight, which was indirectly proved by the improvement of hypothyroidism after bariatric surgery in obese patients (Lips et al., 2013; Ruiz-Tovar et al., 2014; Janssen et al., 2015; Fierabracci et al., 2016). Though the etiology of these changes is still unclear, several other possible mechanisms have been postulated to explain the obesity-hypothyroidism relationship, such as the adaptive process to increase energy expenditure, chronic low-grade inflammation, and abnormal peripheral metabolism of T4/T3 (Durbin-Naltchayan et al., 1983; Pearce, 2012; Santini et al., 2014; Fontenelle et al., 2016). The molecular mechanisms underlying the increased risk of hypothyroidism in obese patients are still not well-defined, and need to be elucidated in future research.

Recent studies suggested that obesity could increase the risk of autoimmune diseases, such as psoriatic arthritis and rheumatoid arthritis, suggesting a critical role of obesity and adipokines in the development of autoimmunity (Russolillo et al., 2013; Gremese et al., 2014; Versini et al., 2014). In our cross-sectional study, though there was lack of an obvious association between obesity and TAI, BMI was significantly correlated with TPOAb in men and BMI was significantly correlated with TGAb in women, which also provided some evidence for the impact of obesity on TAI risk. Nowadays, the impact of obesity on TAI has not been characterized clearly, and more studies are warranted to provide a full evaluation.

Adipose tissues have critical roles in regulating multiple processes, and its dysfunction in obese individuals can thus result in a series of unfavorable outcomes, such as insulin resistance, inflammation, and immune dysfunction (Vieira-Potter, 2014; Alman et al., 2017; Hino et al., 2017). Adipokines are a group of bioactive substances secreted by adipocytes, and hundreds of adipokines have been identified, and adiponectin, leptin, visfatin and resistin are the four most common adipocytes (Freitas Lima et al., 2015). At present, adipokines are regarded as the main players in the interactions between adipose tissues and the immune system (Procaccini et al., 2013; Hutcheson, 2015; Abella et al., 2017). Adipokines can modulate the inflammatory and immune response, and a normal function of adipose tissues is thus critical for maintaining immune tolerance in human bodies (Lourenco et al., 2016; Abella et al., 2017; Danturti et al., 2017). The critical roles of adipokines in modulating immune function provide some explanations for the relationship between obesity and TAI. There is also accumulating evidence from observational studies suggesting that dysregulated adipokines are associated with TAI (Nakamura et al., 2000; Song et al., 2000; Iglesias et al., 2003; Teixeira et al., 2009; Drobniak et al., 2016). However, the molecular mechanisms underlying the roles of obesity and adipokines in the development of TAI are still largely elusive, and more studies are recommended to uncover them and provide new therapeutic targets.

Another important issue is the impact of obesity on the prognosis or progression of hypothyroidism and TAI. Kelderman-Bolk et al. found that higher BMI was associated with poor quality of life in hypothyroidism patients (Kelderman-Bolk et al., 2015). However, there are no other studies available to evaluate whether obesity can aggravate the progression of hypothyroidism and TAI or whether it can affect the treatment outcomes. Therefore, more studies are needed to investigate the roles of obesity on the progression of hypothyroidism and TAI or the treatment outcomes. In addition, the effects of weight loss interventions on thyroid function and thyroid antibodies are also largely elusive, and can be explored in future research.

The prevalence of thyroid disorders including hypothyroidism and TAI vary obviously with age, especially in females. In our study, we analyzed the impact of age on the associations of obesity with hypothyroidism and TAI by conducting subgroup analyses by age of participants (Ages < 65 years vs. ages ≥65 years). Though several distinct associations between participants with different ages were identified (Table 5; Supplementary Table 2), owing to the limited number of participants in our study, the associations of obesity with hypothyroidism and TAI in individuals with different ages were still not well-defined. Therefore, more future studies with large number of participants are necessary to further explore the influence of age on the associations of obesity with hypothyroidism and TAI.

Previous studies have proven that the reference ranges of TSH differ with age, sex, and BMI (Surks and Hollowell, 2007; Boucai et al., 2011; Surks, 2013). Though data from our study suggested that obese individuals didn't have increased level of TSH (*P* > 0.05, Table 2), several previous studies with large number of participants found that obese subjects had increased TSH level and TSH was positively associated with BMI (Asvold et al., 2009; Valdés et al., 2017). Currently, the applied reference range for TSH has been a matter of debate in recent years (Chaker et al., 2017). Most commercially available TSH assays are immunoassays, and the TSH reference range currently used in clinical practice is statistically defined as between the 2.5 and 97.5th percentile in an apparently healthy population (Pearce et al., 2013; Chaker et al., 2017). Though several studies suggested the subpopulation-specific TSH reference ranges could help minimize misclassification (Surks and Hollowell, 2007; Boucai et al., 2011), information on the adequate reference ranges of TSH for each subpopulation is still lacking. Therefore, there is still a generic reference range of TSH for adults with different ages, sex and BMI in clinical practice (Pearce et al., 2013; Chaker et al., 2017). Moreover, owing to the lack of subpopulation-specific TSH reference in our area, we were unable to use subpopulation-specific TSH reference ranges to screen thyroid disorders in the population. Finally, most published studies on the associations of obesity with hypothyroidism and TAI in adults uniformly used a generic reference range of TSH for adults with different ages, sex and BMI (Table 7), which followed current clinical guidelines to define hypothyroidism and SCH (Knudsen et al., 2005; Asvold et al., 2009; Gopinath et al., 2010; Marzullo et al., 2010; Pearce et al., 2013; Jonklaas et al., 2014; Amouzegar et al., 2017; Chaker et al., 2017; Valdés et al., 2017). Therefore, our study also used a generic reference range of TSH to diagnose hypothyroidism and SCH for adults with different ages, sex and BMI. However, the use of subpopulation-specific TSH reference limits can help to better classify patients and minimize misclassification of patients with thyroid dysfunction, and to get a more adequate assessment of the associations of obesity with hypothyroidism and TAI, future studies recruiting larger number of participants and using subpopulation-specific TSH reference ranges are recommended.

**Table 7:** Summary of published studies on the associations of obesity with hypothyroidism and thyroid autoimmunity in adults^*^.

**Study [Ref.]**	**Country**	**Design**	**Participants (Ages, years; Female, %)**	**TSH reference value**	**Adjustment or matching factors**	**Main findings**
Knudsen et al., 2005	Denmark	Cross-sectional	4,082 participants (18–65 years; NA)	0.4–3.6 mIU/L	Age, sex, region of inhabitancy, and smoking.	A significant association between obesity and increasing serum TSH level (>3.6 mIU/L).
Asvold et al., 2009	Norway	Cross-sectional	27,097 participants (>40 years; 67.7%)	0.5–3.5 mIU/L	Age, and smoking.	Significant associations between obesity and SCH both in men (OR = 1.83, 95%CI 1.43–2.35) and women (OR = 1.54, 95%CI 1.34–1.76); A significant association between obesity and overt hypothyroidism in women (OR = 1.66, 95%CI 1.14–2.42).
Gopinath et al., 2010	Australia	Cohort	1,768 participants (≥55 years with as mean age of 67.6 years; 54.4%)	0.1–4.0 mIU/L	Age, and sex.	Significant associations of obesity with hypothyroidism (OR = 2.52, 95%CI 1.29–4.89) and overt hypothyroidism (OR = 4.05, 95%CI 1.74–9.41), but not for SCH (OR = 1.06, 95%CI 0.33–3.37)
Marzullo et al., 2010	Italy	Case-control	165 obese subjects and 118 controls (< 50 years with a mean age of 35.8 years; 59.5%)	0.4–4.0 mIU/L (0.4–4.38 mIU/L for obese patients)	Age and sex.	Significant associations of obesity with hypothyroidism (OR = 3.43, 95%CI 1.35–8.74), but not for SCH (OR = 2.43, 95%CI 0.92–6.41) and overt hypothyroidism (OR = 10.75, 95%CI 0.62–186.5); A significant association between obesity and thyroid autoimmunity (OR = 1.86, 95%CI 1.08–3.21).
Amouzegar et al., 2017	Iran	Cohort	4,204 individuals (≥20 years with as mean age of 40.4 years; 54.4%)	0.34–5.06 mIU/L	Age, sex, smoking, waist circumference, and TPOAb.	Significant associations of obesity with overt hypothyroidism (RR = 2.92, 95%CI 1.64–5.20) and SCH (RR = 1.54, 95%CI 1.14–2.07).
Valdés et al., 2017	Spain	Cross-sectional	3,928 participants (Aged 18–93 years with a mean of 50.0 years; 54.4%)	0.6–5.5 mIU/L	Age, sex, smoking status, and urinary iodine concentrations	Significant association between obesity and SCH (OR = 1.80, 95%CI 1.10–2.92).

* *Studies recruiting children, adolescents, or pregnant women were not reviewed in this table. NA, not available; RR, risk ratio; 95%CI, 95% confidence interval*.

Fat distribution differs by sex, and there is an obvious difference in the body fat depots between women and men, which may cause the sex differences in the associations of obesity with hypothyroidism and TAI (Fried et al., 2015; Palmer and Clegg, 2015). However, the impact of fat distribution on hypothyroidism and TAI is still unclear because studies on this aspect is sill lacking. Future studies are needed to explore the associations of fat distribution with hypothyroidism and TAI by using reliable body composition measurement methods (Fried et al., 2015; Palmer and Clegg, 2015; Lemos and Gallagher, 2017).

Since obesity is positively correlated with diabetes, diabetes might have some influence on the associations of obesity with hypothyroidism and TAI. In our study, we analyzed the possible impact of diabetes on the associations of obesity with hypothyroidism and TAI by conducting subgroup analyses stratified by diabetes (Diabetic participants vs. Non-diabetic participants). Though modest difference existed in the associations of obesity with hypothyroidism and TAI between diabetic participants and non-diabetic participants (Supplementary Tables 1, 2), the limited number of participants in our study impaired the statistical power to adequately detect the possible difference above. Therefore, studies with more participants are needed to further explore the influence of diabetes on the associations of obesity with hypothyroidism and TAI.

Several other limitations should be noted when interpreting the findings of this study. Our study was a cross-sectional study, which could not establish the causality in the associations of obesity with hypothyroidism and TAI. The causative relationship of obesity with hypothyroidism and TAI need to be assessed in future studies using longitudinal data. Besides, we were unable to investigate whether the associations of obesity with hypothyroidism and TAI could be modified by environmental factors, such as dietary patterns and physical exercise. Moreover, the impact of obesity on hyperthyroidism was not analyzed owing to the low number of hyperthyroidism participants. A study by Holm et al. reported that obesity could reduce risk of hyperthyroidism (Holm et al., 2005), but no other study on this aspect was available. More studies are recommended to determine the impact of obesity on hyperthyroidism. Finally, the results from the cross-sectional study could not be generalized to other ethnic populations. The sex differences in the associations of obesity with hypothyroidism and TAI in other ethnic populations are still not well-defined, and our study underscores the need for future researches investigating the sex-specific roles of obesity on hypothyroidism and TAI among other ethnic populations.

In conclusion, the findings in our study highlight sex differences in the associations of obesity with hypothyroidism and TAI among Chinese adults. There is a significant association of obesity with hypothyroidism in women but not in men among Chinese adults. The findings above need verifying in future larger scale studies. Further research is needed to better understand the exact mechanism of sex differences in the obesity-thyroid relationship. Moreover, the exact mechanisms directly connecting obesity with hypothyroidism and TAI remain largely elusive, which need be elucidated in future researches.

## Author contributions

BW and JZ designed the study, collected data, performed statistical analyses, and wrote the final version of the manuscript. RS and WH participated in data collection and performed statistical analyses. QY, QL, and XJ participated in data collection. All authors approved the final version of the manuscript.

### Conflict of interest statement

The authors declare that the research was conducted in the absence of any commercial or financial relationships that could be construed as a potential conflict of interest.
