# Supplementary material for: Sex Differences in the Associations of Obesity With Hypothyroidism and Thyroid Autoimmunity Among Chinese Adults
Source: Front Physiol. 2018 Oct 4;9:1397. doi: 10.3389/fphys.2018.01397 (PMC6180185; doi:10.3389/fphys.2018.01397)
Supplement: Supplementary file 1 [file Table_1.pdf]

Supplementary table 1 Risk of hypothyroidism and thyroid autoimmunity among obese individuals compared with non-obese individuals in the subgroup analyses by diabetes

Thyroid dysfunction	Obese individuals	Non-obese controls	Crude OR		Adjusted OR*	
			OR(95%CI)	P	OR(95%CI)	P
**Non-diabetic participants**						
Total	N = 310	N = 2,129				
Hypothyroidism#	39(12.6%)	263(12.3%)	1.02(0.71-1.46)	0.91	1.20(0.82-1.75)	0.34
SCH	37(11.9%)	242(11.4%)	1.06(0.73-1.53)	0.77	1.24(0.84-1.82)	0.28
TAI	33(10.6%)	278(13.1%)	0.79(0.54-1.16)	0.23	1.01(0.67-1.50)	0.97
TPOAb positivity	28(9.0%)	205(9.6%)	0.93(0.61-1.41)	0.74	1.14(0.74-1.76)	0.54
TGAb positivity	30(9.7%)	242(11.4%)	0.83(0.56-1.24)	0.38	1.14(0.75-1.74)	0.52
Men	N = 201	N = 970				
Hypothyroidism#	15(7.5%)	88(9.1%)	0.81(0.45-1.43)	0.46	0.94(0.52-1.69)	0.83
SCH	14(6.9%)	85(8.7%)	0.78(0.43-1.40)	0.40	0.91(0.49-1.67)	0.76
TAI	15(7.5%)	71(7.3%)	1.02(0.57-1.82)	0.94	1.07(0.59-1.96)	0.81
TPOAb positivity	12(5.9%)	52(5.4%)	1.12(0.59-2.14)	0.73	1.19(0.60-2.34)	0.62
TGAb positivity	13(6.5%)	51(5.2%)	1.25(0.66-2.34)	0.49	1.28(0.66-2.48)	0.47
Women	N = 109	N = 1,159				
Hypothyroidism#	24(22.0%)	175(15.1%)	1.59(0.98-2.57)	0.06	1.50(0.92-2.45)	0.10
SCH	23(21.1%)	157(13.5%)	**1.71(1.04-2.78)**	**0.03**	1.61(0.98-2.66)	0.06
TAI	18(16.5%)	207(17.9%)	0.91(0.54-1.54)	0.72	0.96(0.56-1.65)	0.89
TPOAb positivity	16(14.7%)	153(13.2%)	1.13(0.65-1.97)	0.66	1.13(0.64-1.99)	0.67
TGAb positivity	17(15.6%)	191(16.5%)	0.94(0.54-1.61)	0.81	1.04(0.60-1.81)	0.88
**Diabetic participants**						
Total	N = 87	N = 282				
Hypothyroidism#	16(18.4%)	36(12.8%)	1.54(0.81-2.94)	0.19	1.49(0.76-2.91)	0.24
SCH	16(18.4%)	31(11.0%)	1.82(0.94-3.52)	0.07	1.79(0.91-3.55)	0.09
TAI	16(18.4%)	32(11.3%)	1.76(0.91-3.39)	0.09	1.77(0.89-3.51)	0.10
TPOAb positivity	10(11.5%)	23(8.1%)	1.46(0.67-3.20)	0.34	1.39(0.62-3.12)	0.41
TGAb positivity	12(13.8%)	24(8.5%)	1.72(0.82-3.60)	0.15	1.93(0.87-4.23)	0.10
Men	N = 51	N = 157				
Hypothyroidism#	7(13.7%)	18(11.5%)	1.23(0.48-3.13)	0.67	1.13(0.42-3.06)	0.80
SCH	7(13.7%)	16(10.2%)	1.40(0.54-3.63)	0.48	1.35(0.49-3.68)	0.56
TAI	7(13.7%)	12(7.6%)	1.92(0.71-5.18)	0.19	1.69(0.59-4.80)	0.32
TPOAb positivity	4(7.8%)	10(6.4%)	1.25(0.37-4.17)	0.72	1.04(0.29-3.73)	0.95
TGAb positivity	4(7.8%)	5(3.2%)	2.58(0.67-10.03)	0.17	2.43(0.56-10.43)	0.23
Women	N = 36	N = 125				
Hypothyroidism#	9(25.0%)	18(14.4%)	1.98(0.80-4.89)	0.14	1.95(0.77-4.93)	0.16
SCH	9(25.0%)	15(12.0%)	2.44(0.96-6.18)	0.06	2.36(0.90-6.16)	0.08
TAI	9(25.0%)	20(16.0%)	1.75(0.72-4.27)	0.22	1.91(0.76-4.78)	0.17
TPOAb positivity	6(16.7%)	13(10.4%)	1.72(0.60-4.91)	0.31	1.75(0.59-5.16)	0.31
TGAb positivity	8(22.2%)	19(15.2%)	1.59(0.63-4.02)	0.32	1.81(0.69-4.71)	0.22

(*Confounding factors in the multiple logistic regression analysis included age, sex, smoking, diabetes, hypertension, salt type and urinary iodine concentration. #Hypothyroidism included SCH and overt hypothyroidism. OR, odds ratio; 95%CI, 95% confidence interval.)
