# Supplementary material for: Sex Differences in the Associations of Obesity With Hypothyroidism and Thyroid Autoimmunity Among Chinese Adults
Source: Front Physiol. 2018 Oct 4;9:1397. doi: 10.3389/fphys.2018.01397 (PMC6180185; doi:10.3389/fphys.2018.01397)
Supplement: Supplementary file 2 [file Table_2.pdf]

Supplementary table 2 Correlations between BMI and thyroid autoimmunity in the subgroup analyses by sex or age

Outcomes	Spearman's correlation analysis		Univariate linear regression analysis		Multiple linear regression analysis	
	r(SE)	P value	β(SE)	P value	β(SE)	P value
**Ages <65 years**						
**Total**						
TPOAb	0.015(0.021)	0.447	-0.003(0.007)	0.657	0.004(0.008)	0.599
TGAb	-0.035(0.021)	0.075	**-0.021(0.007)**	**0.005**	-0.003(0.008)	0.711
**Men**						
TPOAb	0.041(0.029)	0.146	0.014(0.007)	0.054	0.012(0.008)	0.144
TGAb	0.018(0.029)	0.514	0.009(0.007)	0.232	0.009(0.008)	0.227
**Women**						
TPOAb	0.022(0.029)	0.435	-0.001(0.013)	0.921	-0.005(0.014)	0.720
TGAb	**-0.057(0.029)**	**0.042**	**-0.028(0.013)**	**0.034**	-0.019(0.014)	0.169
**Ages ≥65 years**						
**Total**						
TPOAb	0.098(0.058)	0.082	0.028(0.024)	0.244	0.025(0.024)	0.306
TGAb	0.054(0.058)	0.337	0.001(0.024)	0.989	-0.005(0.024)	0.846
**Men**						
TPOAb	**0.197(0.082)**	**0.013**	0.046(0.025)	0.061	**0.053(0.025)**	**0.040**
TGAb	0.116(0.082)	0.149	0.005(0.030)	0.864	0.007(0.031)	0.830
**Women**						
TPOAb	0.007(0.082)	0.928	0.007(0.040)	0.861	0.006(0.042)	0.887
TGAb	-0.008(0.082)	0.920	-0.007(0.036)	0.848	-0.008(0.038)	0.824
**Diabetic participants**						
**Total**						
TPOAb	**0.126(0.053)**	**0.015**	**0.041(0.017)**	**0.019**	**0.039(0.018)**	**0.024**
TGAb	**0.124(0.053)**	**0.017**	0.023(0.017)	0.190	0.021(0.018)	0.235
**Men**						
TPOAb	0.126(0.072)	0.068	**0.041(0.017)**	**0.017**	**0.045(0.018)**	**0.012**
TGAb	**0.172(0.072)**	**0.013**	**0.025(0.012)**	**0.044**	0.026(0.013)	0.051
**Women**						
TPOAb	0.137(0.081)	0.084	0.040(0.032)	0.211	0.035(0.033)	0.289
TGAb	0.070(0.081)	0.378	0.021(0.036)	0.560	0.019(0.037)	0.613
**Non-diabetic participants**						
**Total**						
TPOAb	0.018(0.021)	0.361	-0.003(0.008)	0.663	0.001(0.008)	0.919
TGAb	-0.035(0.021)	0.080	**-0.022(0.008)**	**0.004**	-0.008(0.008)	0.316
**Men**						
TPOAb	0.054(0.030)	0.065	0.014(0.008)	0.079	0.011(0.008)	0.194
TGAb	0.023(0.030)	0.435	0.008(0.008)	0.338	0.007(0.009)	0.443
**Women**						
TPOAb	0.012(0.029)	0.666	-0.001(0.013)	0.977	-0.011(0.014)	0.451
TGAb	**-0.061(0.029)**	**0.029**	**-0.034(0.013)**	**0.014**	-0.026(0.014)	0.075

(Values of thyroid antibodies were nonnormally distributed, and were log2-transformed in the linear regression analysis. SE, standard error.)
